# Crystal Structure of the SPOC Domain of the *Arabidopsis* Flowering Regulator FPA

**DOI:** 10.1371/journal.pone.0160694

**Published:** 2016-08-11

**Authors:** Yinglu Zhang, Katarzyna Rataj, Gordon G. Simpson, Liang Tong

**Affiliations:** 1 Department of Biological Sciences, Columbia University, New York, NY, 10027, United States of America; 2 Division Plant Sciences & Centre for Gene Regulation & Expression, School of Life Sciences, University of Dundee, Dundee, DD1 5EH, Scotland, United Kingdom; 3 Cell & Molecular Sciences, The James Hutton Institute, Invergowrie, Dundee, Scotland, United Kingdom; Universidad Miguel Hernández de Elche, SPAIN

## Abstract

The *Arabidopsis* protein FPA controls flowering time by regulating the alternative 3′-end processing of the *FLOWERING LOCUS* (*FLC*) antisense RNA. FPA belongs to the split ends (SPEN) family of proteins, which contain N-terminal RNA recognition motifs (RRMs) and a SPEN paralog and ortholog C-terminal (SPOC) domain. The SPOC domain is highly conserved among FPA homologs in plants, but the conservation with the domain in other SPEN proteins is much lower. We have determined the crystal structure of *Arabidopsis thaliana* FPA SPOC domain at 2.7 Å resolution. The overall structure is similar to that of the SPOC domain in human SMRT/HDAC1 Associated Repressor Protein (SHARP), although there are also substantial conformational differences between them. Structural and sequence analyses identify a surface patch that is conserved among plant FPA homologs. Mutations of two residues in this surface patch did not disrupt FPA functions, suggesting that either the SPOC domain is not required for the role of FPA in regulating RNA 3′-end formation or the functions of the FPA SPOC domain cannot be disrupted by the combination of mutations, in contrast to observations with the SHARP SPOC domain.

## Introduction

Eukaryotic messenger RNAs (mRNAs) are made as precursors through transcription by RNA polymerase II (Pol II), and these primary transcripts undergo extensive processing, including 3′-end cleavage and polyadenylation [1,2,3,4]. In addition, alternative 3′-end cleavage and polyadenylation is an essential and ubiquitous process in eukaryotes [5,6]. Misregulation of (alternative) 3′-end processing can lead to various genetic defects, cancer and other diseases [7,8]. There is currently great interest in understanding the molecular mechanisms and functional impacts of alternative 3′-end processing.

**Fig 1:**
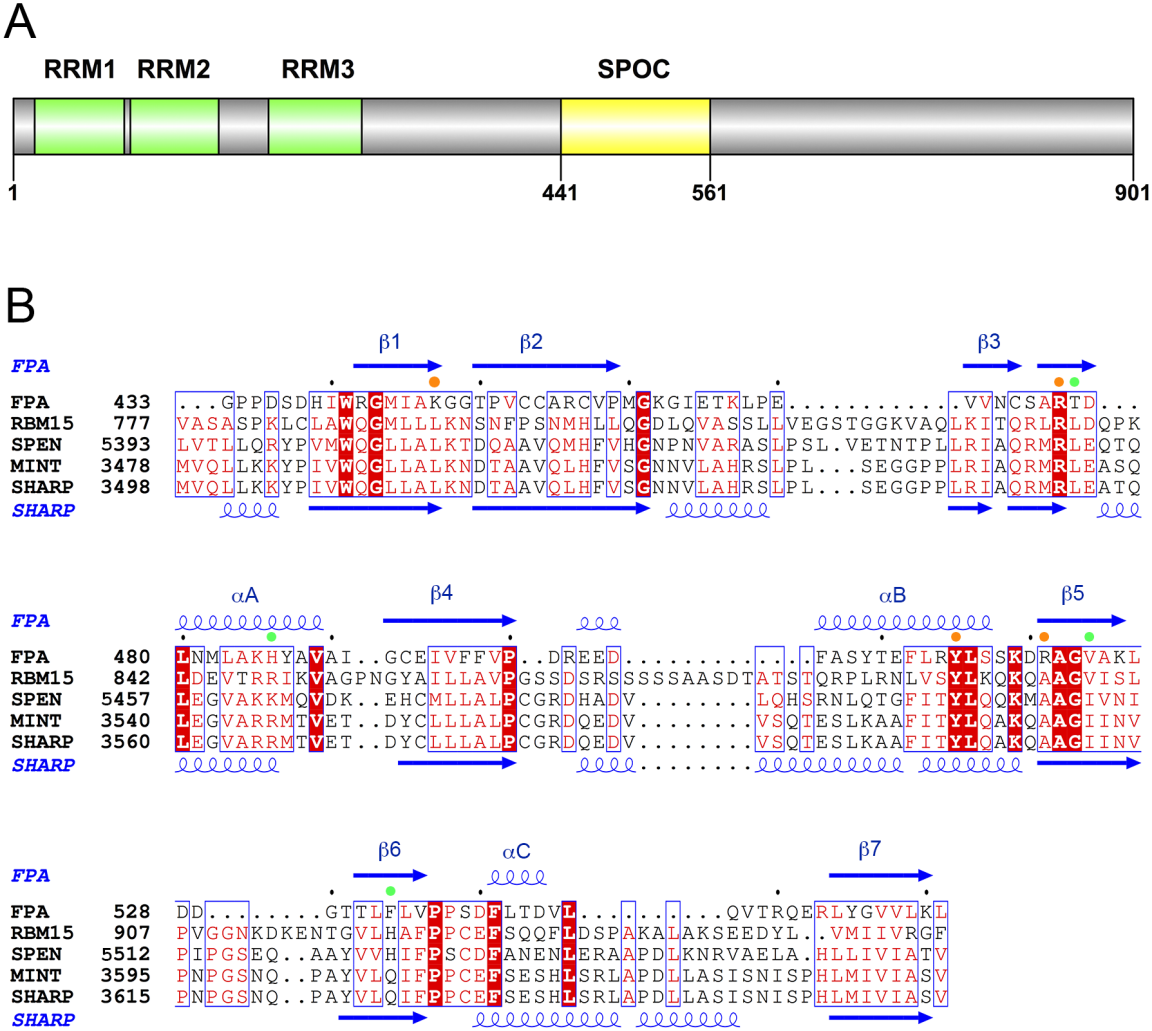
Sequence conservation of SPOC domains. **(A)**. Domain organization of *A*. *thaliana* FPA. **(B)**. Sequence alignment of the SPOC domains of *Arabidopsis thaliana* FPA, human RBM15, *Drosophila* SPEN, mouse MINT, and human SHARP. Residues in surface patch 1 are indicated with the orange dots, and those in surface patch 2 with the green dots. The secondary structure elements in the structure of FPA SPOC are labeled. Residues that are strictly conserved among the five proteins are shown in white with a red background, and those that are mostly conserved in red.

Recently, the split ends (SPEN) family of proteins was identified as RNA binding proteins that regulate alternative 3′-end cleavage and polyadenylation [9]. They are characterized by possessing N-terminal RNA recognition motifs (RRMs) and a conserved SPEN paralog and ortholog C-terminal (SPOC) domain [10,11] (Fig 1A). The SPOC domain is believed to mediate protein-protein interactions and has diverse functions among SPEN family proteins, but the molecular mechanism of these functions is not well understood.

FPA, a SPEN family protein in *Arabidopsis thaliana* and other plants, was found to regulate the 3′-end alternative cleavage and polyadenylation of the antisense RNAs of *FLOWERING LOCUS* (*FLC*), a flowering repressor gene [12,13,14]. FPA promotes the 3′-end processing of class I *FLC* antisense RNAs, which includes the proximal polyadenylation site. This is associated with histone demethylase activity and down-regulation of *FLC* transcription. However, the functional mechanism of this complex is still not clear.

Although a SPOC domain is found in all the SPEN family proteins, its sequence conservation is rather low. For example, the sequence identity between the SPOC domains of *A*. *thaliana* FPA and human SMRT/HDAC1 Associated Repressor Protein (SHARP) is only 19% (Fig 1B). Currently, the SHARP SPOC domain is the only one with structural information [15,16].

As a first step toward understanding the molecular basis for the regulation of alternative 3′-end processing and flowering by FPA, we have determined the crystal structure of the SPOC domain of *A*. *thaliana* FPA at 2.7 Å resolution. The overall structure is similar to that of the SHARP SPOC domain, although there are also substantial conformational differences between them. The structure reveals a surface patch that is conserved among FPA homologs.

## Results and Discussion

### Structure of FPA SPOC domain

The crystal structure of the SPOC domain of *A*. *thaliana* FPA has been determined at 2.7 Å resolution using the selenomethionyl single-wavelength anomalous dispersion method. The expression construct contained residues 433–565 of FPA, but only residues 439–460 and 465–565 are ordered in the crystal. The atomic model has good agreement with the X-ray diffraction data and the expected bond lengths, bond angles and other geometric parameters (Table 1). All the residues are located in the favored regions of the Ramachandran plot (data not shown). The structure has been deposited in the Protein Data Bank, with accession code 5KXF.

**Table 1:** Summary of crystallographic information.

Resolution range (Å)^1^	50–2.7 (2.8–2.7)
Number of observations	78,008
*R*_merge_ (%)	10.5 (45.3)
I/σI	24.1 (6.3)
Redundancy	
Completeness (%)	100 (100)
*R* factor (%)	19.2 (25.0)
Free *R* factor (%)	25.4 (35.4)
Rms deviation in bond lengths (Å)	0.017
Rms deviation in bond angles (°)	1.9

^1^The numbers in parentheses are for the highest resolution shell.

**Fig 2:**
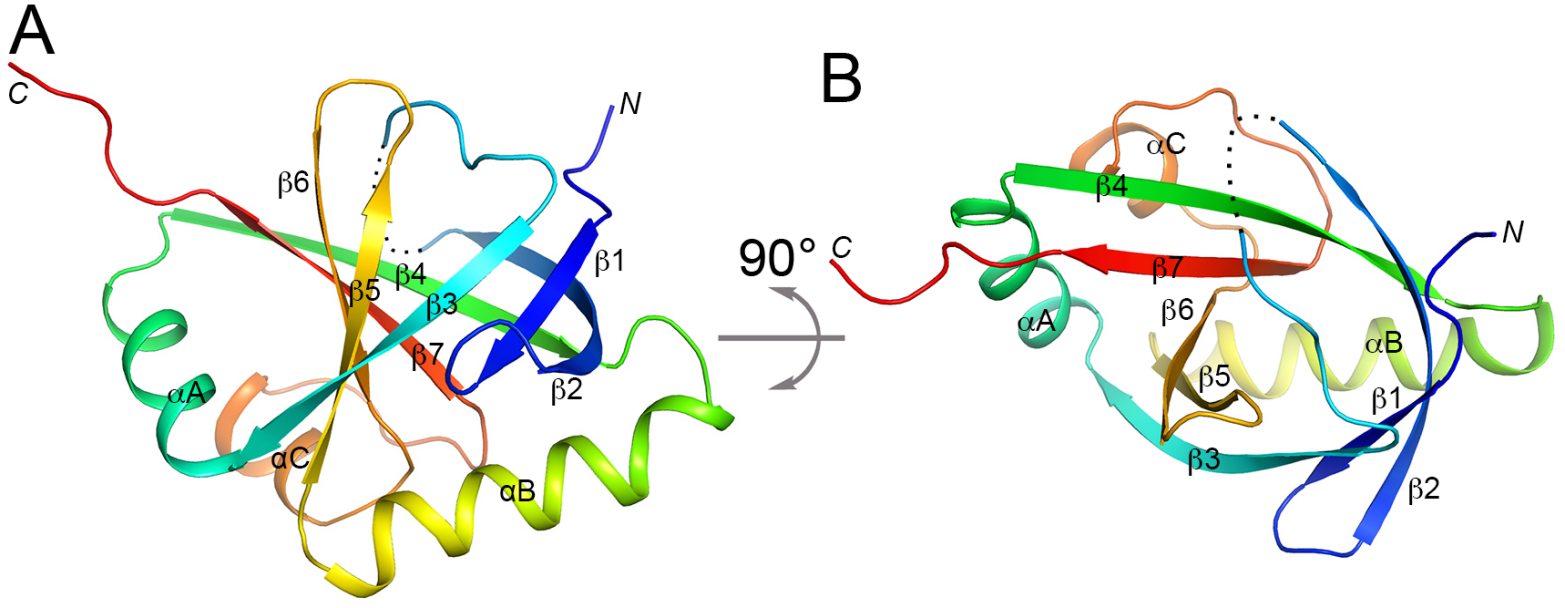
Crystal structure of the SPOC domain of *A*. *thaliana* FPA. **(A)**. Schematic drawing of the structure of FPA SPOC domain, colored from blue at the N terminus to red at the C terminus. The view is from the side of the β-barrel. The disordered segment (residues 460–465) is indicated with the dotted line. (**B**). Structure of the FPA SPOC domain, viewed from the end of the β-barrel, after 90° rotation around the horizontal axis from panel A. All structure figures were produced with PyMOL (www.pymol.org).

The crystal structure of the FPA SPOC domain contains a seven-stranded, mostly anti-parallel β-barrel (β1-β7) and three helices (αA-αC) (Fig 2A). Only two of the neighboring strands, β1 and β3, are parallel to each other. Helix αB covers one end of the barrel, while helices αA and αC are located next to each other at one side of the barrel (Fig 2B). The other end of the β-barrel is covered by the loop connecting strands β2 and β3, which contains the disordered 461–464 segment. The center of the barrel is filled with hydrophobic side chains and is not accessible to the solvent.

### Comparisons to structural homologs of the SPOC domain

**Fig 3:**
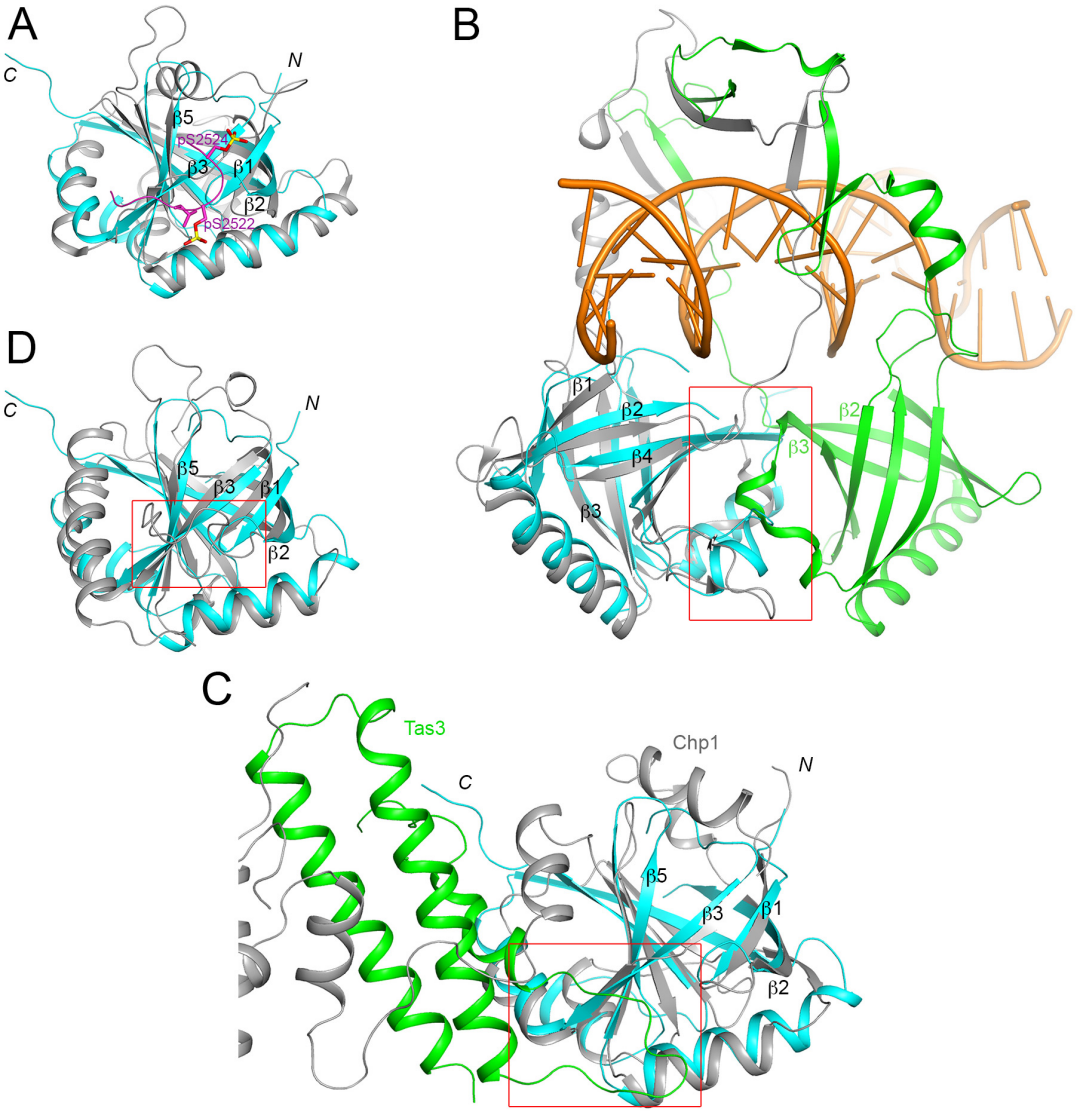
Structural homologs of the FPA SPOC domain. **(A)**. Overlay of the structures of the FPA SPOC domain (cyan) and the SHARP SPOC domain (gray). The bound position of a doubly-phosphorylated peptide from SMRT is shown in magenta. **(B)**. Overlay of the structures of the FPA SPOC domain (cyan) and the Ku70 β-barrel domain (gray). Ku80 contains a homologous domain (green), which forms a hetero-dimer with that in Ku70. The two domains, and inserted segments on them, mediate the binding of dsDNA (orange). The red rectangle highlights the region of contact between the two β-barrel domains. **(C)**. Overlay of the structures of the FPA SPOC domain (cyan) and the homologous domain in Chp1 (gray). The binding partner of Chp1, Tas3, is shown in green. The red rectangle indicates the region equivalent to the binding site of the SMART phosphopeptide in SHARP SPOC domain, where a loop of Tas3 is also located. (**D**). Overlay of the structures of the FPA SPOC domain (cyan) and the Med25 ACID (gray).

Only five structural homologs of the FPA SPOC domain were found in the Protein Data Bank with the DaliLite server [17], suggesting that the SPOC domain structure is relatively unique. The top hit is the SPOC domain of human SHARP [15,16] (Fig 3A), with a *Z* score of 12.3. The other four structural homologs include the β-barrel domain of the proteins Ku70 and Ku80 (*Z* score 11.4) [18] (Fig 3B), a domain in the chromodomain protein Chp1 (*Z* score 10.8) [19] (Fig 3C), and the activator interacting domain (ACID) of the Med25 subunit of the Mediator complex (*Z* score 8.5) [20,21,22,23] (Fig 3D). The next structural homolog has a *Z* score of 3.0.

SHARP is a transcriptional co-repressor in the nuclear receptor and Notch/RBP-Jκ signaling pathways [24,25]. The SPOC domain of SHARP interacts directly with silencing mediator for retinoid and thyroid receptor (SMRT), nuclear receptor co-repressor (N-CoR), HDAC, and other components to represses transcription. While the overall structure of the FPA SPOC domain is similar to that of the SHARP SPOC domain, there are noticeable differences in the positioning of the β-strands and the helices, and most of the loops have substantially different conformations as well (Fig 3A). In addition, the SHARP SPOC domain has three extra helices. One of them covers the other end of the β-barrel, and the other two shield an additional surface of the side of the β-barrel from solvent. A doubly-phosphorylated peptide from SMRT is bound to the side of the barrel, near strands β1 and β3 [16] (Fig 3A). Such a binding mode probably would not be possible in FPA, as the peptide would clash with the β1-β2 loop.

The Ku70-Ku80 hetero-dimer is involved in DNA double-strand break repair and the β-barrel domain contributes to DNA binding [18]. In fact, the β-barrel domains of Ku70 and Ku80 form a hetero-dimer, primarily through interactions between the loops connecting the third and fourth strands of the barrel (Fig 3B). The open ends of the two β-barrels face the DNA binding sites, and contact the phosphodiester backbone of the dsDNA. In addition, a long insert connecting strands β2 and β3 in the two domains form an arch-like structure, encircling the dsDNA.

Chp1 is a subunit of the RNA-induced initiation of transcriptional gene silencing (RITS) complex [19]. The partner of Chp1, Tas3, is bound between the barrel domain and the second domain of Chp1, and the linker between the two domains is also crucial for this interaction (Fig 3C). It is probably unlikely that the β-barrel itself is sufficient to bind Tas3. Interestingly, a loop in Tas3 contacts strand β3 of the barrel domain, at a location somewhat similar to that of the N-terminal segment of the SMRT peptide in complex with SHARP SPOC domain (Fig 3A).

Mediator is a coactivator complex that promotes transcription by Pol II. The Med25 subunit ACID is the target of the potent activator VP16 of the herpes simplex virus [20,21,22,23]. The structure of ACID contains a helix at the C-terminus as well as an extended β1-β2 loop. Nonetheless, the binding site for VP16 has been mapped to roughly the same surface patch, near strands β1 and β3, that is used by the SHARP and Tas3 SPOC domains for binding their partners.

### A conserved surface patch in the FPA SPOC domain

**Fig 4:**
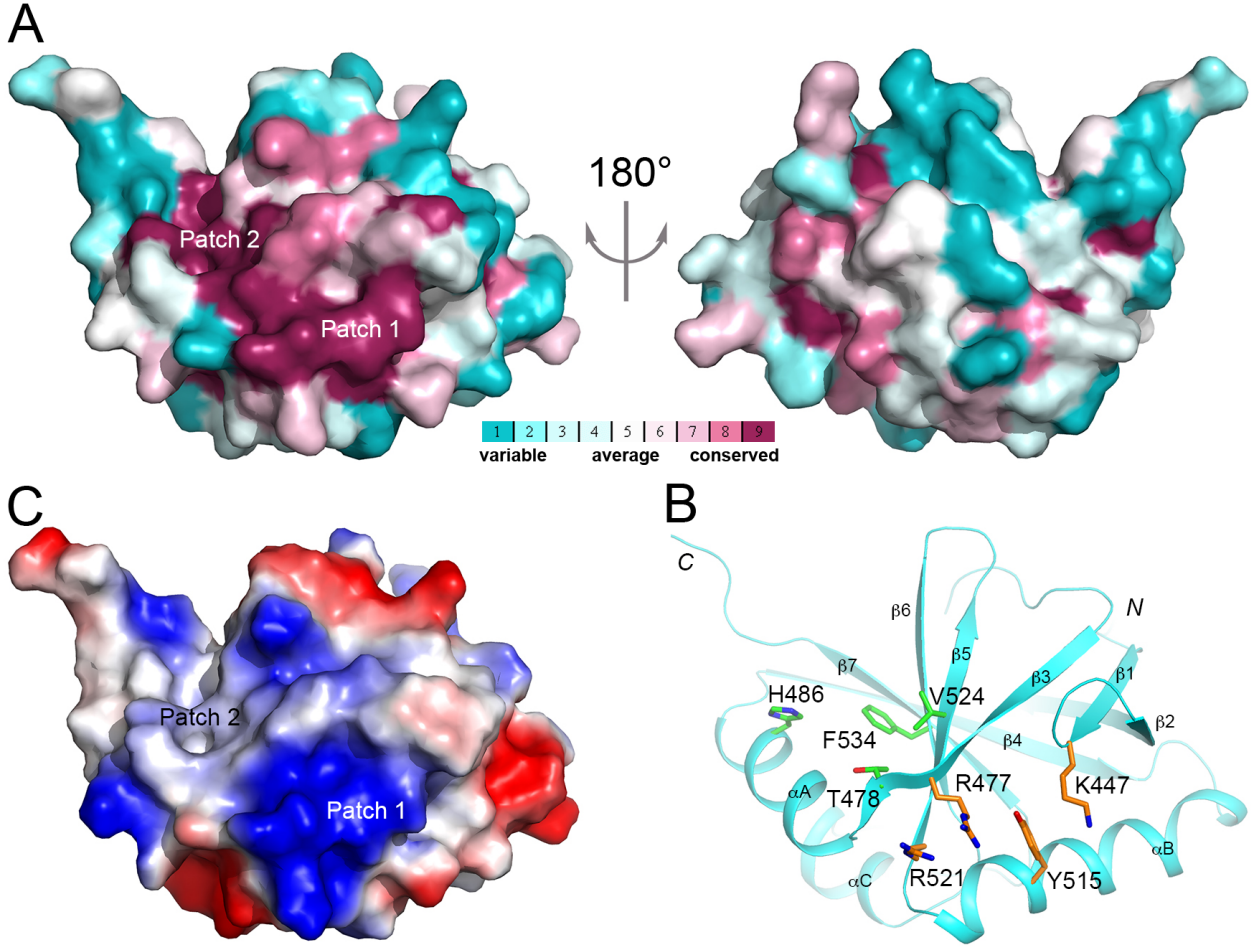
A conserved surface patch of FPA SPOC domain. **(A)**. Two views of the molecular surface of FPA SPOC domain colored based on sequence conservation among plant FPA homologs. Purple: most conserved; cyan: least conserved. **(B)**. Residues in the conserved surface patch of FPA SPOC domain. The side chains of the residues are shown in stick models, colored orange in the first sub-patch and green in the second. **(C)**. Molecular surface of FPA SPOC domain colored based on electrostatic potential. Blue: positively charged; red: negatively charged.

An analysis of the SPOC domain indicates a large surface patch near strands β1, β3, β5 and β6 that is conserved among plant FPA homologs (Fig 4A) [26]. This surface patch can be broken into two sub-patches, with residues Lys447 (in strand β1), Arg477 (β3), Tyr515 (αB) and Arg521 (β5) in one sub-patch, and residues His486 (αA), Thr478 (β3), Val524 (β5) and Phe534 (β6) in the other sub-patch (Fig 4B). The first surface patch is electropositive in nature (Fig 4C), and residues Arg477 and Tyr515 are also conserved in the SHARP SPOC domain (Fig 1B). In fact, one of the phosphorylated residues of the SMRT peptide interacts with this surface patch (Fig 3A), suggesting that the FPA SPOC domain might also interact with a phosphorylated segment here. In comparison, the second surface patch is more hydrophobic in nature (Fig 4C).

### Testing the requirement of specific conserved amino acids for FPA functions

We next examined the potential impact of the conserved surface patch on FPA function *in vivo*. We mutated two residues, Arg477 and Tyr515, of the surface patch, which are also conserved in the SHARP SPOC domain (Fig 1B) and were found to be functionally important [15]. The mutations were introduced into a transgene designed to express FPA from its native control elements (promoter, introns and 3′ UTR). The resulting transgenes were then stably transformed into an *fpa-8* mutant background so that the impact of the mutations on FPA function could be assessed. Control transformation of the same expression constructs into *fpa-8* designed to express wild-type FPA protein restored FPA protein expression levels to near wild-type levels (panel A in S1 Fig) and rescued the function of FPA in controlling RNA 3′-end formation, for example in FPA pre-mRNA (panel B in S1 Fig). We examined independent transgenic lines expressing each R477A and Y515A mutation. In each case, we confirmed that detectable levels of FPA protein expression were restored close to wild-type levels in protein blot analyses using antibodies that specifically recognize FPA (S2 Fig).

**Fig 5:**
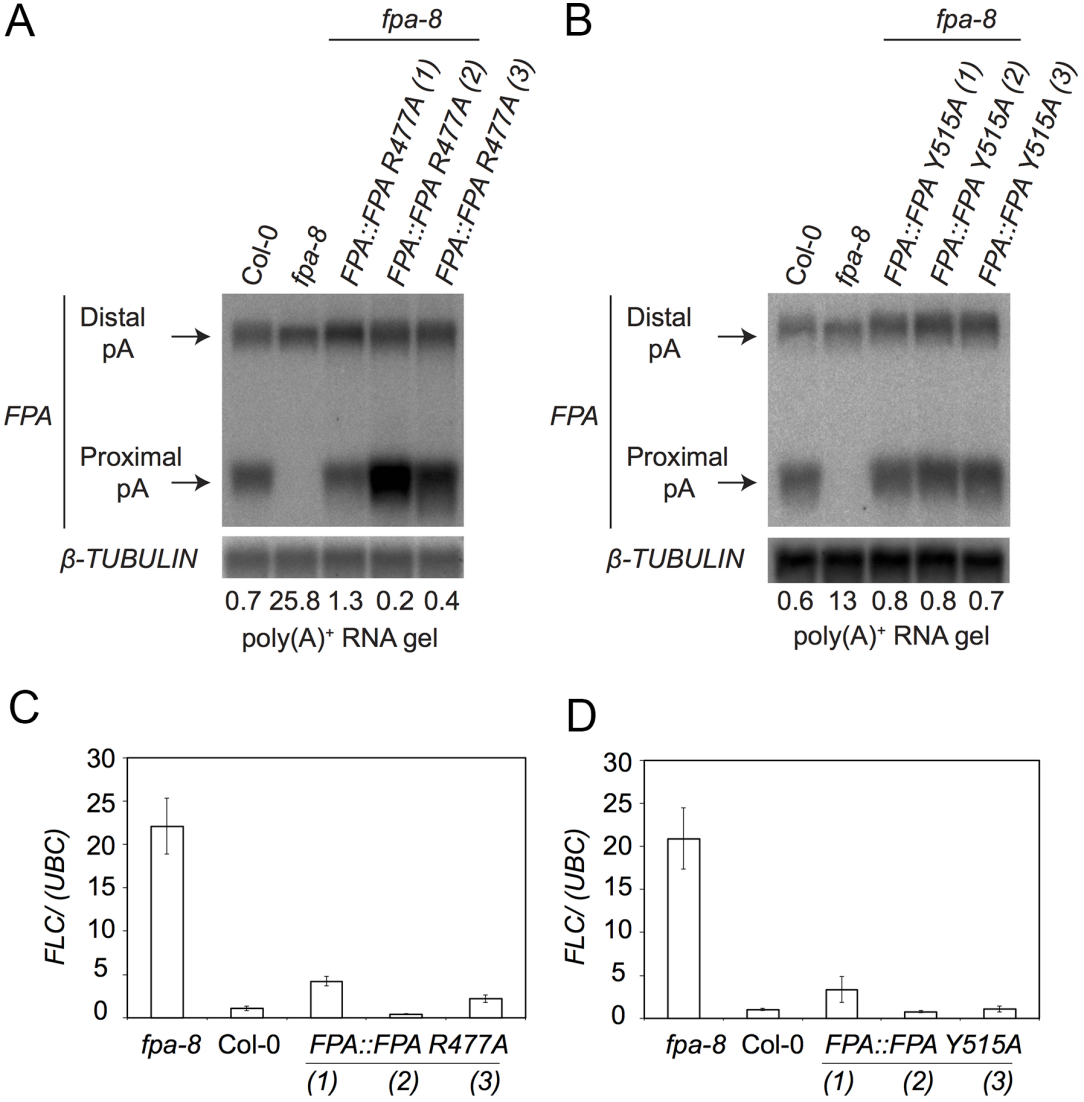
Impact of individual FPA SPOC domain mutations on alternative polyadenylation of FPA pre-mRNA. RNA gel blot analysis of WT *A*. *thaliana* accession Columbia (Col-0) plants *fpa-8* and *fpa-8* mutants expressing either *FPA*::*FPA R477A* **(A)**, or *FPA*::*FPA Y515A* **(B)** using poly(A)+ purified mRNAs. A probe corresponding to the 5’UTR region of *FPA* mRNA was used to detect *FPA* specific mRNAs. RNA size (kb) marker (Ambion). *TUBULIN* was detected as an internal control. Proximally and distally polyadenylated *FPA* transcripts are marked with arrows. The ratio of distal:proximal polyadenylated forms is given under each lane. **(C,D)** Impact of individual FPA SPOC domain mutations on *FLC* transcript levels. qRT-PCR analysis was performed with total RNA purified from Col-0, *fpa-8*, *35S*::*FPA*:*YFP* and *FPA*::*FPA R477A* **(C)**, *FPA*::*FPA Y515A* **(D)** plants. Transcript levels were normalized to the control *UBC*. Histograms show mean values ±SE for three independent PCR amplifications of three biological replicates.

We then examined the impact of the surface patch mutations on FPA’s function in controlling RNA 3′-end formation by determining whether the mutant proteins functioned in *FPA* autoregulation [13] and the repression of *FLC* expression [12]. FPA autoregulates its expression by promoting cleavage and polyadenylation within intron 1 of its own pre-mRNA, resulting in a truncated transcript that does not encode functional protein [13]. We used RNA gel blot analyses to reveal that in each of three independent transgenic lines for each single mutant, rescue of proximally polyadenylated *FPA* pre-mRNA can be detected (Fig 5A and 5B). We therefore conclude that neither of these mutations disrupted the ability of FPA to promote RNA 3′-end formation in its own transcript.

We next examined whether the corresponding mutations disrupted the ability of FPA to control *FLC* expression. We used RT-qPCR to measure the expression of *FLC* mRNA and found that in each independent transgenic line encoding each mutated FPA protein, the elevated levels of *FLC* detected in *fpa-8* mutants were restored to near wild-type levels by expression of the FPA SPOC conserved patch mutant proteins (Fig 5C and 5D).

**Fig 6:**
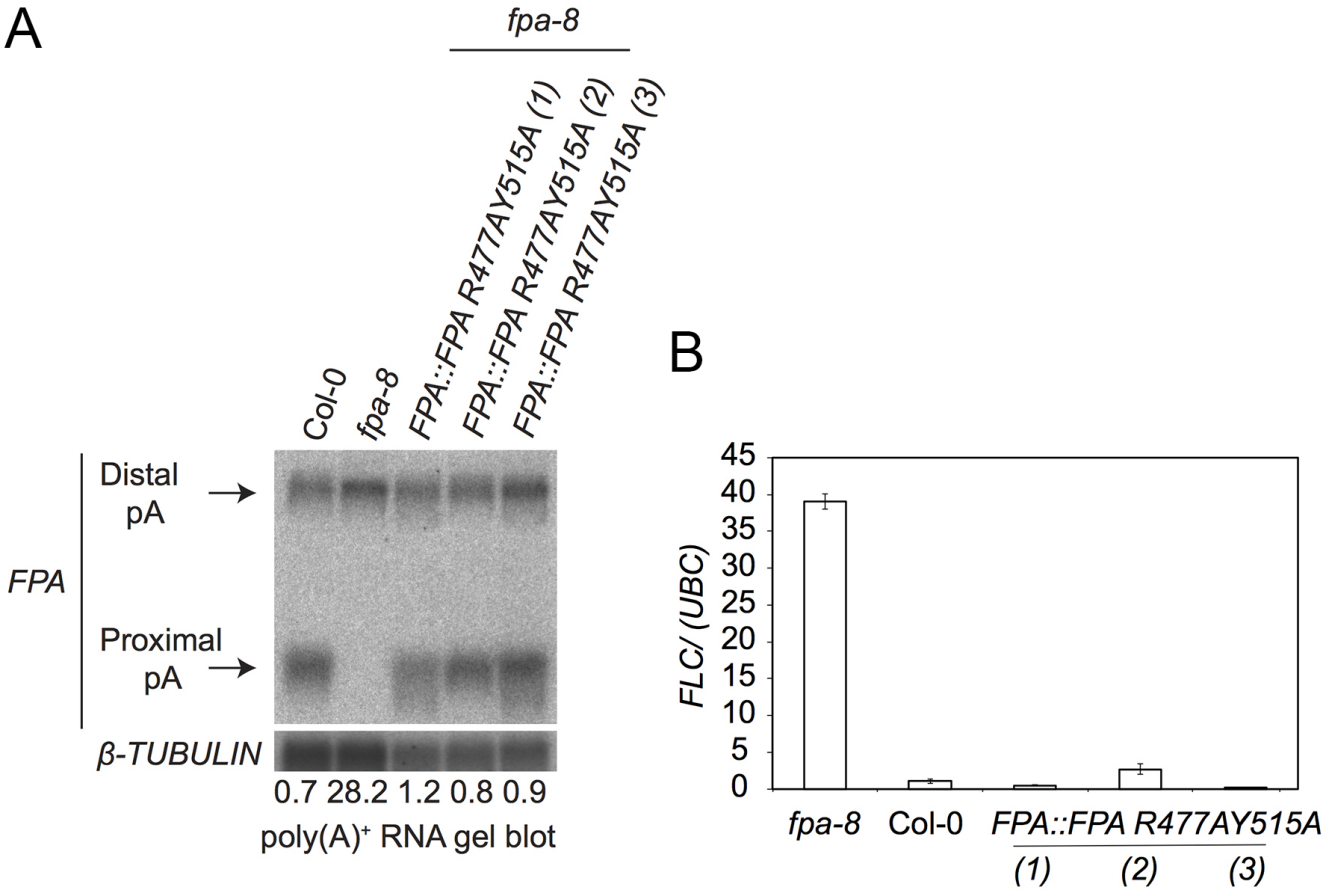
Impact of double FPA SPOC domain mutations on alternative polyadenylation of FPA pre-mRNA and *FLC* expression. **(A)** RNA gel blot analysis of WT *A*. *thaliana* accession Columbia (Col-0) plants *fpa-8* and *fpa-8* mutants expressing *FPA*::*FPA R477A;Y515A* using poly(A)+ purified mRNAs. Black arrows indicate the proximally and distally polyadenylated *FPA* mRNAs. A probe corresponding to the 5’UTR region of *FPA* mRNA was used to detect *FPA* specific mRNAs. RNA size (kb) marker (Ambion). *TUBULIN* was detected as an internal control. The ratio of distal:proximal polyadenylated forms is given under each lane. **(B)**. qRT-PCR analysis was performed with total RNA purified from Col-0, *fpa-8*, and *FPA*::*FPA R477A;Y515A* plants. Transcript levels were normalized to the control *UBC*. Histograms show mean values ±SE for three independent PCR amplifications of three biological replicates.

Since each surface patch mutation appeared to be insufficient to disrupt FPA functions on its own, we combined both mutations into the same transgene. We could again confirm that near wild-type levels of FPA protein were expressed from three independent transgenic lines expressing the FPA R477A;Y515A doubly mutated protein in an *fpa-8* mutant background (S3 Fig). We found that FPA R477A;Y515A protein functioned like wild-type FPA to restore *FPA* pre-mRNA proximal polyadenylation (Fig 6A) and *FLC* expression to wild-type levels (Fig 6B).

Together our findings suggest that either the SPOC domain is not required for the role of FPA in regulating RNA 3′-end formation, or that this combination of mutations is not sufficient to critically disrupt the function of the FPA SPOC domain. Since the corresponding mutations in the SHARP SPOC domain do disrupt its recognition of unphosphorylated SMRT peptides [16], these observations may reinforce the idea that the features and functions of the FPA SPOC domain differ from those of the only other well-characterized SPOC domain.

## Materials and Methods

### Protein expression and purification

The SPOC domain (residue 433–565) of *A*. *thaliana* FPA was sub-cloned into the pET28a vector (Novagen). The recombinant protein, with an N-terminal hexa-histidine tag, was over-expressed in *E*. *coli* BL21 Star (DE3) cells (Novagen), which were induced with 0.4 mM IPTG and allowed to grow at 20°C for 14–18 h. The soluble protein was purified by nickel-charged immobilized-metal affinity chromatography and gel filtration chromatography. The purified protein was concentrated and stored at –80°C in a buffer containing 20 mM Tris (pH 8.0), 200 mM NaCl, 10 mM DTT and 5% (v/v) glycerol. The His-tag was not removed for crystallization.

The selenomethionine labeled SPOC domain was expressed in *E*. *coli* B834(DE3) strain using LeMaster media [27] and purified with the same protocol as the native protein.

### Protein crystallization

Crystals of the native SPOC domain of FPA were grown at 20°C with the sitting-drop vapor diffusion method. The protein solution was at 30 mg/ml concentration, and the reservoir solution contained 0.2 M MgSO_4_, and 20% (v/v) PEG 3350. Fully-grown crystals were obtained two days after set-up. Crystals of the selenomethionine labeled SPOC domain were grown using the same condition as the native protein. The crystals were cryo-protected in the crystallization solution supplemented with 20% (v/v) glycerol and flash-frozen in liquid nitrogen for data collection at 100K.

### Data collection and processing

A single-wavelength anomalous dispersion (SAD) X-ray diffraction data set on a selenomethionine labeled SPOC domain crystal was collected at the National Synchrotron Light Source (NSLS) beamline X29A using an ADSC Q315r CCD. The diffraction images were processed and scaled with the HKL package [28]. The crystal belongs to space group *P*6_5_, with unit cell parameters of *a* = *b* = 108.2 Å, and *c* = 34.2 Å.

### Structure determination and refinement

The structure of the SPOC domain was solved by the selenomethionyl SAD method [29] with the program SHELX [30]. The phases were used by program PHENIX [31] for automatic model building. Manual model rebuilding was carried out with Coot [32]. The structure refinement was performed with the program PHENIX, with translation, libration, and screw-rotation (TLS) parameters. The data processing and refinement statistics are summarized in Table 1. The Ramachandran plot showed that 95.8% of the residues are located in the most favored regions, and 4.2% are in additional allowed regions.

### Generation of constructs with mutated genomic FPA sequence

A series of constructs containing a mutated FPA genomic sequence was prepared based on pGreen I 0029 vector [33]. pGreen I 0029 vector with inserted FPA genomic sequence was prepared. In this vector FPA genomic sequence is flanked by 2620bp of the native sequence upstream to the start codon and 1178bp downstream to the stop codon. The vector contains kanamycin resistance genes for both the bacteria and plant hosts. In order to obtain a series of constructs with mutated FPA genomic sequence, FPA sequence in this construct was modified using site-directed mutagenesis. Primers used to prepare required constructs are listed in S1 Table. After the mutagenesis reaction the presence of only the desired mutations was confirmed by sequencing of the whole FPA genomic sequence and flanking regions.

### Generation of Arabidopsis thaliana transgenic plants

All transgenic plants were prepared in *fpa-8* mutant background, which is in Col-0 accession [34]. The prepared vectors for *Arabidopsis* transformations were introduced into electro-competent *Agrobacterium tumefaciens* cells (C58 CV3101 strain harbouring pSoup vector). The floral dip method [35] was used for plant transformation. Transgenic plants were selected using kanamycin as a selection marker. Presence of the desired mutations in plants was confirmed with specific dCaps markers.

### Plant growth conditions

Wild type Col-0 plants used in this study were obtained from the Nottingham Arabidopsis Stock Centre. Seed of *fpa-8* and *35S*::*FPA*:*YFP* were obtained from Professor Caroline Dean. Plants were grown in pots containing Universal Extra general purpose soil. The glasshouse temperature was maintained at 20°C and the 16 hour daylight was provided by high pressure sodium vapour lamps (Philips Powertone SON-T AGRO 400). In order to grow plants in sterile conditions, seeds were first surface sterilized by a 5 min treatment with sterilizing solution (3% v/v sodium hypochlorite, 0.02% v/v Triton X-100), followed by three washes with 0.02% v/v Triton X-100 and one wash with sterile water. The sterile seeds were sown on MS10 media supplemented with 0.8% w/v agar. MS10 medium was also supplemented with specific antibiotics if required. After sowing, the seeds were stratified at 4°C for two days in order to synchronize their germination. Plants were grown in the tissue culture room at the following conditions: temperature 22°C, 16 hours daylight provided by the Master TL-D 36W/840 (Philips) lamps.

### Plant protein analysis

Total protein samples were prepared using extraction buffer containing: 40 mM Tris-HCl, pH 6.8; 0.1 mM EDTA, pH 8.0; 8 M urea; 1.43 M β-mercaptoethanol, 7% v/v Complete Protease Inhibitors (Roche) and 5 mM PMSF. Equal volumes of samples were separated on 8% SDS-PAGE. Proteins were transferred onto Protran nitrocellulose transfer membrane (Whatman) using wet Criterion blotter system (BioRad). The transfer was performed at room temperature for two hours at a stable voltage of 70 V. Membrane was blocked in 3% (w/v) Milk in TBS for 1h at room temperature followed by overnight incubation with anti-FPA antibody [12] (dilution 1:100 in 3% (w/v) Milk in TBS). After washes the membrane was incubated for 75 min with goat anti-rabbit antibody (Thermo Scientific) (1:3000 dilution in 3% (w/v) Milk in TBS). Protein was detected using SuperSignal^®^ West Femto Maximum Sensitivity Substrate (Thermo Scientific). Blots were re-probed following treatment with low pH solution (25mM glycine-HCl, pH 2, 1% (w/v) SDS) followed by blocking for 1h at room temperature in 3% (w/v) Milk in TBS. The membrane was incubated overnight with anti-TUBB2A, tubulin, beta 2A antibody (ARP40177_P050 Aviva systems biology; (dilution 1:1000 in 3% (w/v) Milk in TBS). After washes the membrane was incubated for 75 min with goat anti-rabbit antibody (Thermo Scientific) [1:3000 dilution in 3% (w/v) Milk in TBS]. Signal was detected using SuperSignal^®^ West Femto Maximum Sensitivity Substrate (Thermo Scientific).

### RNA gel blot analysis and RT-qPCR

RNA gel blot analysis and RT-qPCR method performed as previously described [12].

## Supporting Information

S1 Fig FPA protein and RNA levels. (A). FPA protein level in *FPA*::*FPAwt fpa-8* plants. Proteins isolated from wild type Col-0, *fpa-8*, *35S*::*FPA*:*YFP* and independent *FPA*::*FPAwt* transgenic lines were separated on 8% SDS-PAGE. Western blot analysis was performed with FPA antibody [13]. TUBULIN was detected as a control. (B). RNA gel blot analysis of WT *A*. *thaliana* accession Columbia (Col-0) plants *fpa-8* and *fpa-8* mutants expressing *FPA*::*FPAwt* using poly(A)+ purified mRNAs. A probe corresponding to the 5′UTR region of *FPA* mRNA was used to detect *FPA* specific mRNAs. RNA size (kb) marker (Ambion). *TUBULIN* was detected as an internal control. Proximally and distally polyadenylated *FPA* transcripts are marked with arrows. The ratio of distal:proximal polyadenylated forms is given under each lane. (C). RNA gel blot analysis of *FLC* transcript in *FPA*::*FPAwt* plants. Poly(A)^+^ RNA was isolated from Col-0, *fpa-8* and trangenic lines expressing *FPA*::*FPAwt* in an *fpa-8* mutant background and detected with a probe recognizing *FLC* sequence. *TUBULIN* was detected as a control.
(PDF)

S2 Fig FPA protein level in *FPA*::*FPA R477A* and *FPA*::*FPA Y515A* plants. Proteins isolated from wild type Col-0, *fpa-8*, *35S*::*FPA*:*YFP*, *FPA*::*FPA R477A* (A) and *FPA*::*FPA Y515A* (B) plants were separated on SDS-PAGE. Western blot analysis was performed with FPA antibody. For the loading control TUBULIN antibody was used.
(PDF)

S3 Fig FPA protein level in *FPA*::*FPA R477Y515A* plants. Proteins isolated from wild type Col-0, *fpa-8*, *35S*::*FPA*:*YFP*, *FPA*::*FPA R477AY515A* plants were separated on SDS-PAGE. Western blot analysis was performed with FPA antibody. For the loading control TUBULIN antibody was used.
(PDF)

S1 Table Site-directed mutagenesis primers. (PDF)
