# Supplementary figures and images for: Crystal Structure of the SPOC Domain of the Arabidopsis Flowering Regulator FPA
Source: PLoS One. 2016 Aug 11;11(8):e0160694. doi: 10.1371/journal.pone.0160694 (PMC4981400; doi:10.1371/journal.pone.0160694)

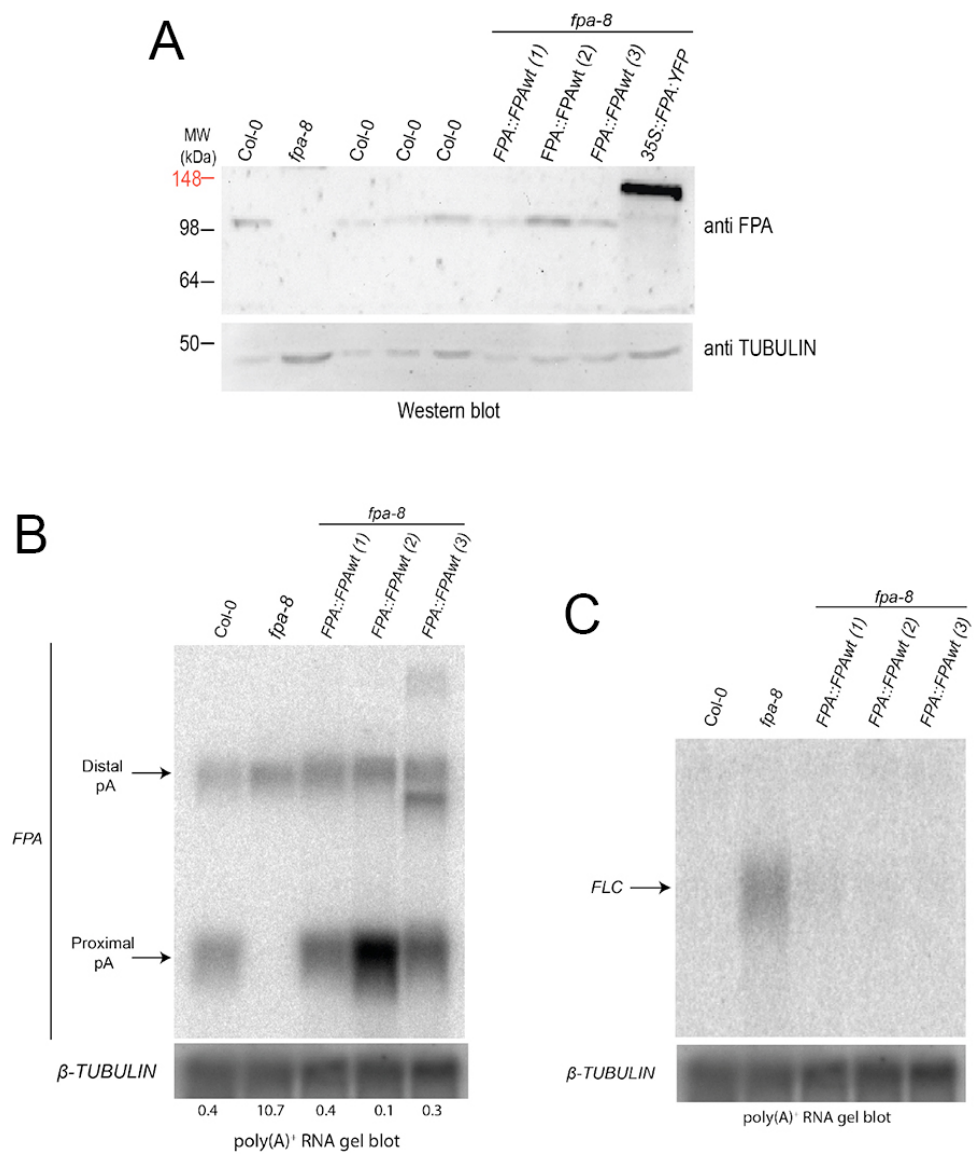

**S1 Figure**

Supplement: S1 Fig — (A). FPA protein level in FPA::FPAwt fpa-8 plants. Proteins isolated from wild type Col-0, fpa-8, 35S::FPA:YFP and independent FPA::FPAwt transgenic lines were separated on 8% SDS-PAGE. Western blot analysis was performed with FPA antibody [13]. TUBULIN was detected as a control. (B). RNA gel blot analysis of WT A. thaliana accession Columbia (Col-0) plants fpa-8 and fpa-8 mutants expressing FPA::FPAwt using poly(A)+ purified mRNAs. A probe corresponding to the 5′UTR region of FPA mRNA was used to detect FPA specific mRNAs. RNA size (kb) marker (Ambion). TUBULIN was detected as an internal control. Proximally and distally polyadenylated FPA transcripts are marked with arrows. The ratio of distal:proximal polyadenylated forms is given under each lane. (C). RNA gel blot analysis of FLC transcript in FPA::FPAwt plants. Poly(A)+ RNA was isolated from Col-0, fpa-8 and trangenic lines expressing FPA::FPAwt in an fpa-8 mutant background and detected with a probe recognizing FLC sequence. TUBULIN was detected as a control. (PDF) [file pone.0160694.s001.pdf]

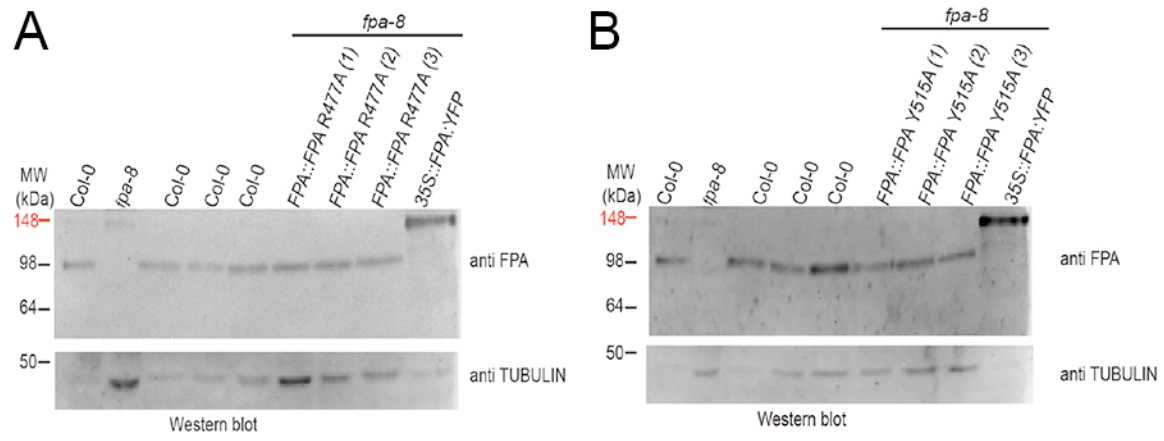

**S2 Figure.**

Supplement: S2 Fig — Proteins isolated from wild type Col-0, fpa-8, 35S::FPA:YFP, FPA::FPA R477A (A) and FPA::FPA Y515A (B) plants were separated on SDS-PAGE. Western blot analysis was performed with FPA antibody. For the loading control TUBULIN antibody was used. (PDF) [file pone.0160694.s002.pdf]

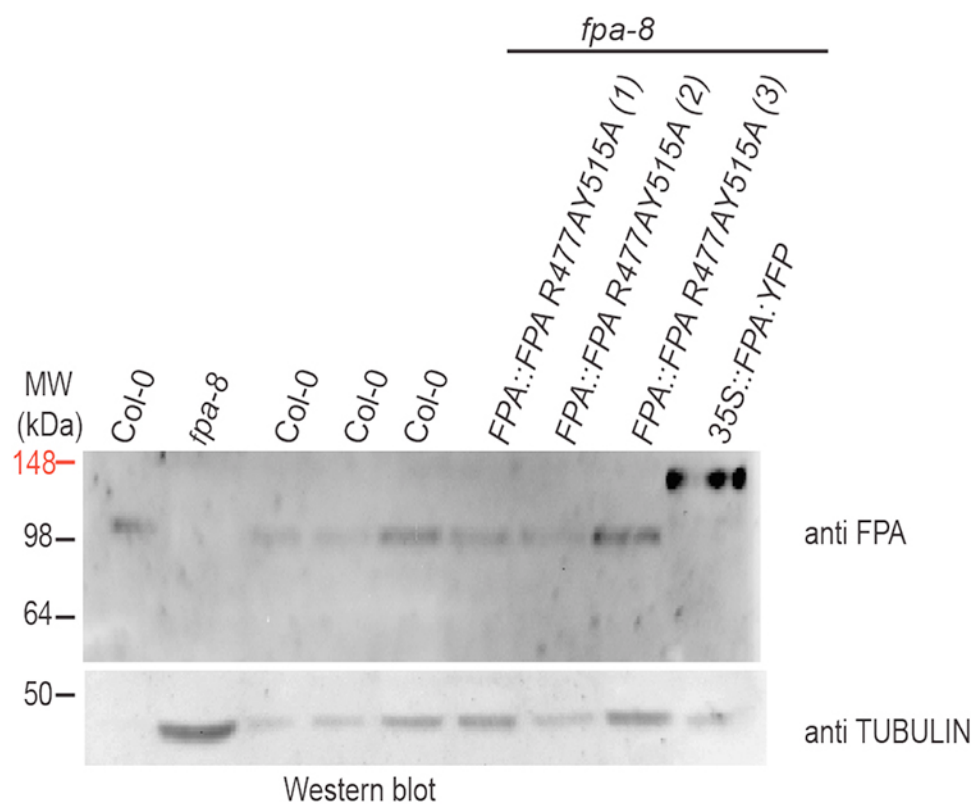

**S3 Figure.**

Supplement: S3 Fig — Proteins isolated from wild type Col-0, fpa-8, 35S::FPA:YFP, FPA::FPA R477AY515A plants were separated on SDS-PAGE. Western blot analysis was performed with FPA antibody. For the loading control TUBULIN antibody was used. (PDF) [file pone.0160694.s003.pdf]
