# Supplementary material for: Crystal Structure of the SPOC Domain of the Arabidopsis Flowering Regulator FPA
Source: PLoS One. 2016 Aug 11;11(8):e0160694. doi: 10.1371/journal.pone.0160694 (PMC4981400; doi:10.1371/journal.pone.0160694)
Supplement: S1 Table — (PDF) [file pone.0160694.s004.pdf]

S1 Table.

Name of the final plasmid	Name of the original plasmid	Primer orientation	Primer sequence (5’ – 3’ orientation)
pGreenI 0029 FPA R477A	*pGreenI 0029 FPA*	sense	CTGAGGTCGTCAATTGTTCAGCAGCGACTGATTTGAATATGCTCGCT
		antisense	AGCGAGCATATTCAAATCAGTCGCTGCTGAACAATTGACGACCTCAG
pGreenI 0029 FPA Y515A	*pGreenI 0029 FPA*	sense	TCTTACACTGAATTTCTCCGGGCGCTTAGCTCAAAAGATCGGGCG
		antisense	CGCCCGATCTTTTGAGCTAAGCGCCCGGAGAAATTCAGTGTAAGA
pGreenI 0029 FPA R477A Y515A	*pGreenI 0029 FPA Y515A*	sense	CTGAGGTCGTCAATTGTTCAGCAGCGACTGATTTGAATATGCTCGCT
		antisense	AGCGAGCATATTCAAATCAGTCGCTGCTGAACAATTGACGACCTCAG

Primers were used to generate a range of pGreen I 0029 vectors with mutated *FPA* genomic sequence. Name of the final clone and the vector used in the mutagenesis reaction as well as sequence of sense and antisense primers are included.
